# Differential responses to aging amongst the transcriptome and proteome of mesenchymal progenitor populations

**DOI:** 10.21203/rs.3.rs-3755129/v1

**Published:** 2023-12-15

**Authors:** Gustavo Duque, Jack Feehan, Nicholas Tripodi, Dmitry Kondrikov, Tissa Wijeratne, Jeffrey Gimble, William Hill, Vasso Apostolopoulos

**Affiliations:** McGill University; Victoria University; Victoria University; The Medical University of South Carolina; University of Melbourne; Tulane University School of Medicine; The Medical University of South Carolina; Victoria University

**Keywords:** Stem cells, mesenchymal stem and progenitor cells, mesenchymal stem cells, circulating osteoprogenitors, adipose-derived stem cells, aging, ageing

## Abstract

The biological aging of mesenchymal stem cells is proposed to contribute to the development of a range of musculoskeletal and systemic diseases associated with older adults, such as osteoporosis, sarcopenia, and frailty. Despite this, little is understood about the specific mechanisms which drive this stem cell exhaustion, with most studies evaluating indirect effects of other aging changes, such as DNA damage, senescence, and inflammaging. In this study, we assess the transcriptomic and proteomic changes in three different populations of mesenchymal progenitor cells from older (50–70 years) and younger (20–40 years) individuals to uncover potential mechanisms driving stem cell exhaustion in mesenchymal tissues. To do this, we harvested primary bone marrow mesenchymal stem and progenitor cells (MPCs), circulating osteoprogenitors (COP), and adipose-derived stem cells (ADSCs) from younger and older donors, with an equal number of samples from males and females. These samples underwent RNA sequencing and label-free proteomic analysis, comparing the younger samples to the older ones. There was a distinct transcriptomic phenotype associated with the pooled older stem cells, indicative of suppressed proliferation and differentiation; however, there was no consistent change in the proteome of the cells. Older MPCs had a distinct phenotype in both the transcriptome and proteome, again consistent with altered differentiation and proliferation, but also a pro-inflammatory immune shift in older adults. COP cells showed a strong transcriptomic shift to pro-inflammatory signaling but no consistent proteomic phenotype. Similarly, ADSCs displayed transcriptomic shift in physiologies associated with cell migration, adherence, and immune activation, but no consistent proteomic change with age. These results show that there are underlying transcriptomic changes with stem cell aging that likely contribute to a decline in tissue regeneration; however, contextual factors such as the microenvironment and general health status also have a strong role in this.

## Introduction

All mesenchymal tissues, such as bone, fat, muscle, and cartilage, undergo a constant process of repair and renewal over the lifespan ^1^. In most instances, this regeneration is driven by a balance between anabolic development and catabolic breakdown of the tissue. As an individual ages, however, these processes are commonly dysregulated, with the balance between anabolism and catabolism lost. This is seen in many tissues and leads to some of the most important aging-related diseases, including osteoporosis, sarcopenia, and osteoarthritis ^2, 3^. The modern field of geroscience seeks to develop a unifying paradigm of physiological changes that occur in aging, which drive the onset of these diseases ^4^. Geroscience has described key physiologies, such as inflammaging, proteostasis, genetic damage, and epigenetic changes as collectively underpinning these diseases ^5, 6^. In addition, another key pillar in musculoskeletal conditions and mesenchymal tissues is stem cell exhaustion ^7^, which is a process by which stem cells lose or alter their multipotency and are less able to maintain tissue quality with increasing age.

Mesenchymal tissue regeneration is mediated by a range of progenitor cells, of which the best understood is the mesenchymal stem and progenitor cells population (MPC), which resides in the perivascular niche in the bone marrow ^1^. Once known as mesenchymal stem cells (MSCs), and thought to be a distinct entity, they are now more accurately characterized as a heterogenous population, though the roles of the sub-populations are poorly understood ^8–10^. Additionally, in recent years, cells with a capacity for mesenchymal differentiation have been identified in various tissues, including the circulation and adipose tissue. The adipose-derived adult stem cell (ADASC) is a plastic adherent, mesenchymal lineage restricted stem cell population derived from brown and white adipose tissue ^11^. These cells are similar in many regards to the bone marrow MPCs, both phenotypically and functionally, but are also involved in regulating energy metabolism due to their localization to adipose tissue. The circulating osteoprogenitor (COP) cell is a newly discovered mesenchymal progenitor in the peripheral circulation of adults ^9, 10^. Once thought to simply be a bone marrow MPC stimulated to circulate, following the discovery of the bone marrow as their origin through parabiosis experiments ^12^, they have since been shown to be phenotypically distinct bearing markers of the hematopoietic lineage. While now known to be a specific population of cells, they have been shown to differentiate along mesodermal lineages and contribute to a number of important diseases, including osteoporosis ^13^, fracture healing ^14^, and vascular calcification ^15^. While there remain significant unknowns about these progenitor populations, they have generated substantial interest in the field of regenerative medicine, with several of them under investigation for therapeutic use in several disease settings, including osteoporosis, sarcopenia, and fracture. However, given these conditions most commonly onset in older age, there is a great need to understand how they are affected by aging.

While MPCs, ADASCs and COP cells have been associated with both physiological and pathological tissue development, there are still significant unknowns regarding their relationships and specific roles. It has been shown that all three change with age, contributing to the development of musculoskeletal diseases. For example, MPCs isolated from older individuals have a decreased capacity to form bone, an increased capacity for adipogenesis, and altered miRNA and epigenetic regulation ^16, 17^, COP cell numbers are associated with age, bone density, and vitamin D status in older adults ^13, 18^, and ADASCs become pro-oxidative, and pro-inflammatory in older adults ^19^. While these physiological outcomes have been shown, there is little understanding of the specific mechanisms driving these changes. It is also unclear whether there is a common physiological driver of these changes across all adult stem cell populations, or whether each group of progenitors is impacted differently. Therefore, we sought to contrast these three groups of progenitors with samples taken from younger and older adults to identify and compare the genetic and proteomic alterations in stem and progenitor cell aging.

## Results

### Differential expression of genes, but not proteins associated with differentiation and proliferation in stem cells isolated from older individuals.

**Figure 1:**
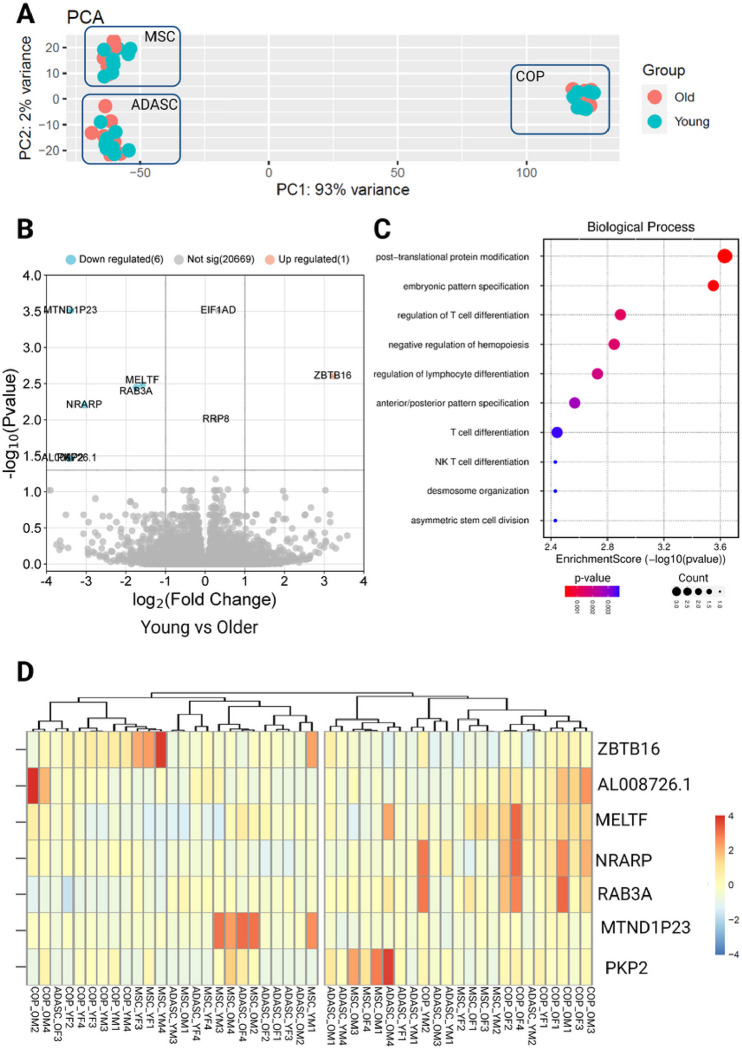
Differential expression of genes across cells grouped by age. A) PCA plot showing clustering by cell type, rather than age, B) Volcano plot showing differential expression of younger samples, compared to older, C) Biological process enrichment analysis, D) Heatmap showing differentially expressed genes. COP: Circulating osteoprogenitor cells; MPC: Mesenchymal stem and progenitor cells; ADASC: Adipose derived adult stem cells; YM: Young Male; YF: Young Female; OM: Older Male; OF: Older Female.

**Figure 2:**
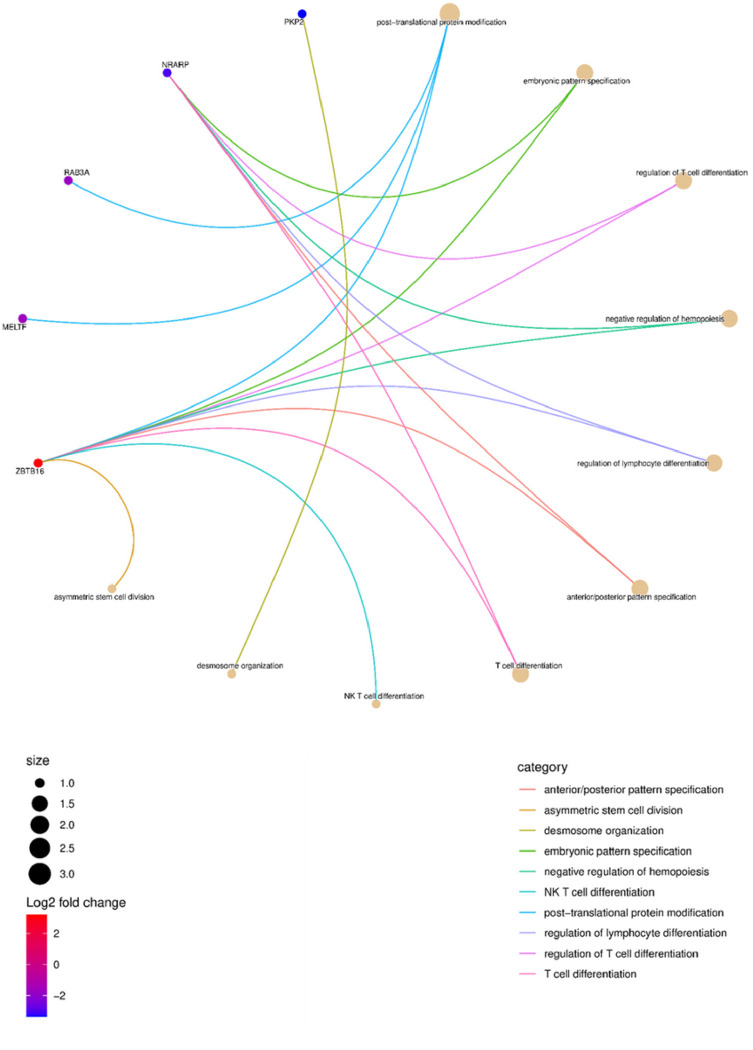
Circular network plot showing the differentially regulated genes, and their biological process ontologies.

In principle component analyses based on the transcriptome, the samples clustered by cell type rather than age, with distinct localization of MPC, ADASC, and COP cells (Fig. 1A). Differential expression analyses revealed seven differentially expressed genes (DEGs) common across the three cell types (Fig. 1B and D). Pathway and ontology analysis of the DEGs showed significant enrichment in pathways associated with differentiation, immune activation, stem cell division, and embryonic pattern specification (Fig. 1C, Fig. 2). None of these translational changes were preserved at a proteome level, with no differentially expressed proteins across the age groups.

### Differential expression of genes and proteins in MPCs from older and younger donors.

**Figure 3:**
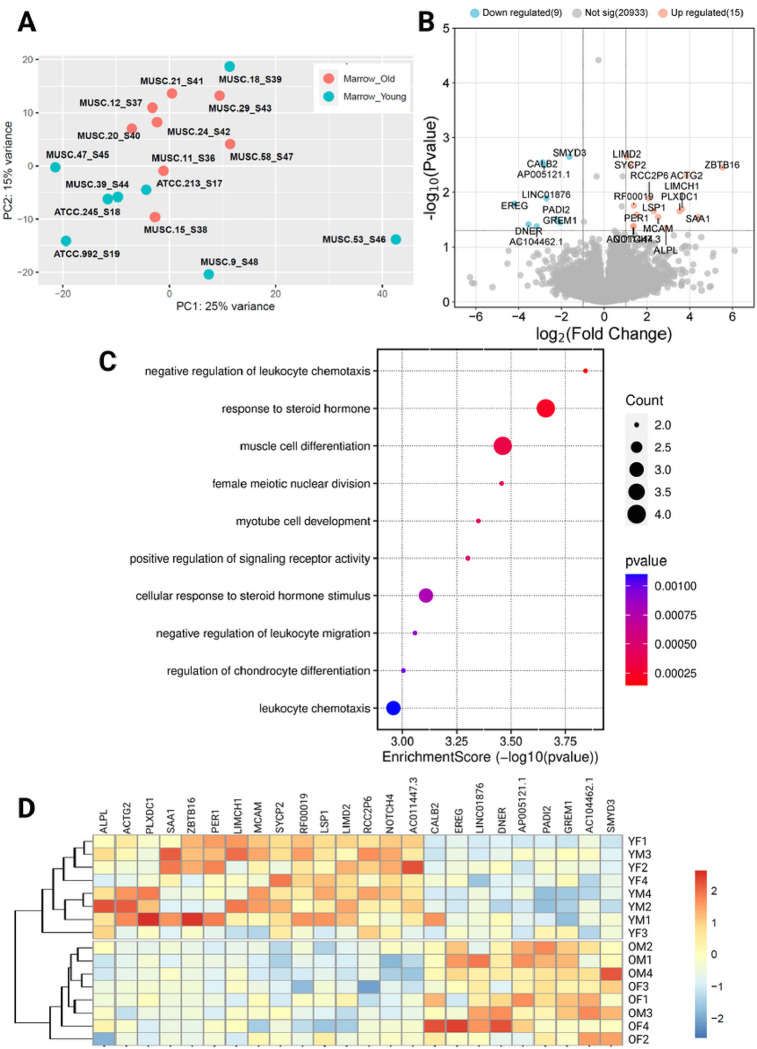
Transcriptome analysis of MPCs. A) PCA plot showing limited clustering of samples by age, B) Volcano plot showing differentially expressed genes in younger donors vs older, C) Biological process ontology enrichment analysis of differentially expressed genes, and D) Clustered heat map showing differentially expressed genes across the data set. COP: Circulating osteoprogenitor cells; MPC: Mesenchymal stem and progenitor cells; ADASC: Adipose derived adult stem cells; YM: Young Male; YF: Young Female; OM: Older Male; OF: Older Female.

**Figure 4:**
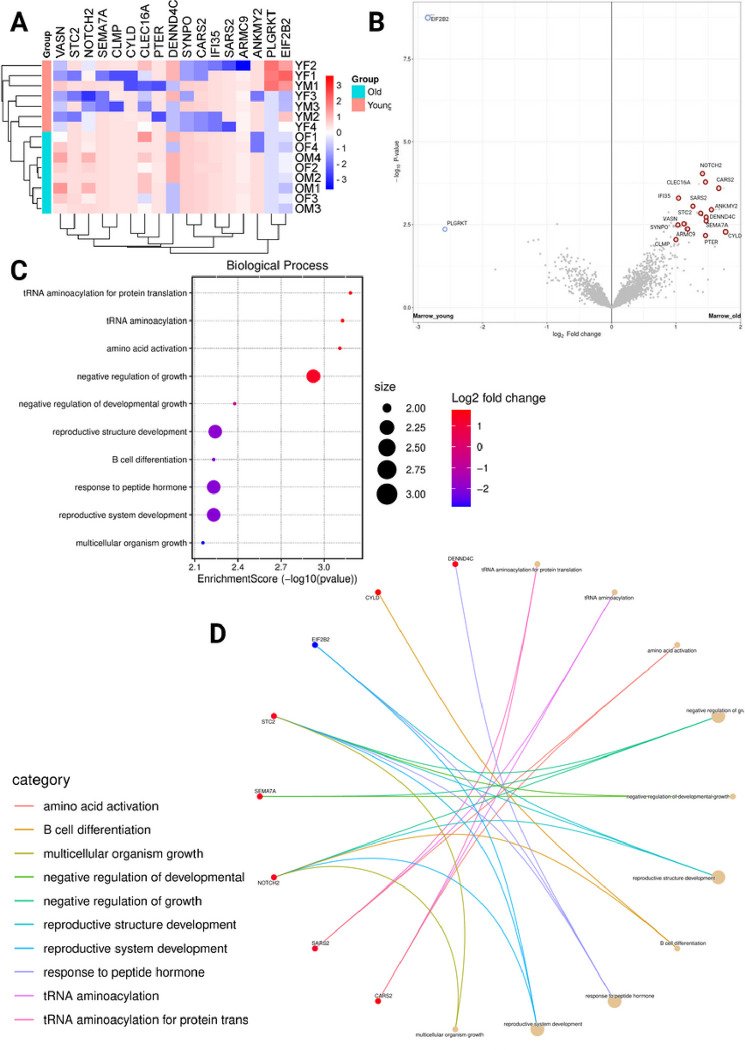
Proteomic analysis of MPCs A) Heatmap showing differentially expressed proteins between older and younger donors, B) Volcano plot showing differentially expressed proteins between younger and older, C) Biological process ontology analysis showing enrichment of pathways in the set, D) Circular network plot showing proteins and their associations with the enriched pathways. COP: Circulating osteoprogenitor cells; MPC: Mesenchymal stem and progenitor cells; ADASC: Adipose derived adult stem cells; YM: Young Male; YF: Young Female; OM: Older Male; OF: Older Female.

Analysis of the bone marrow MPCs alone showed greater differential expression between older and younger samples, with both transcriptomic and proteomic changes. In principle component analyses, there was limited clustering of cells along any dimension (Fig. 3A). There was a total of 24 DEGs across the two age groups, with 9 genes under-expressed in younger samples and 15 genes over-expressed (Fig. 3B and D). Biological process ontology analysis showed significant enrichment of pathways associated with muscle differentiation and development, cell division, response to steroid hormones, regulation of chondrocyte differentiation, and immune system activation and migration (Fig. 3C). At a protein level, there were 17 differentially expressed proteins (Fig. 4A), with 2 proteins under-expressed and 15 over-expressed in older donors vs younger donors (Fig. 4B). Biological process ontology analysis revealed enrichment of genes associated with the regulation of growth, tRNA aminoacylation, and hormone responses in the differentially expressed protein set (Fig. 4C and D).

### Differentially expressed genes, but not proteins in COP cells and ADSCs.

**Figure 5:**
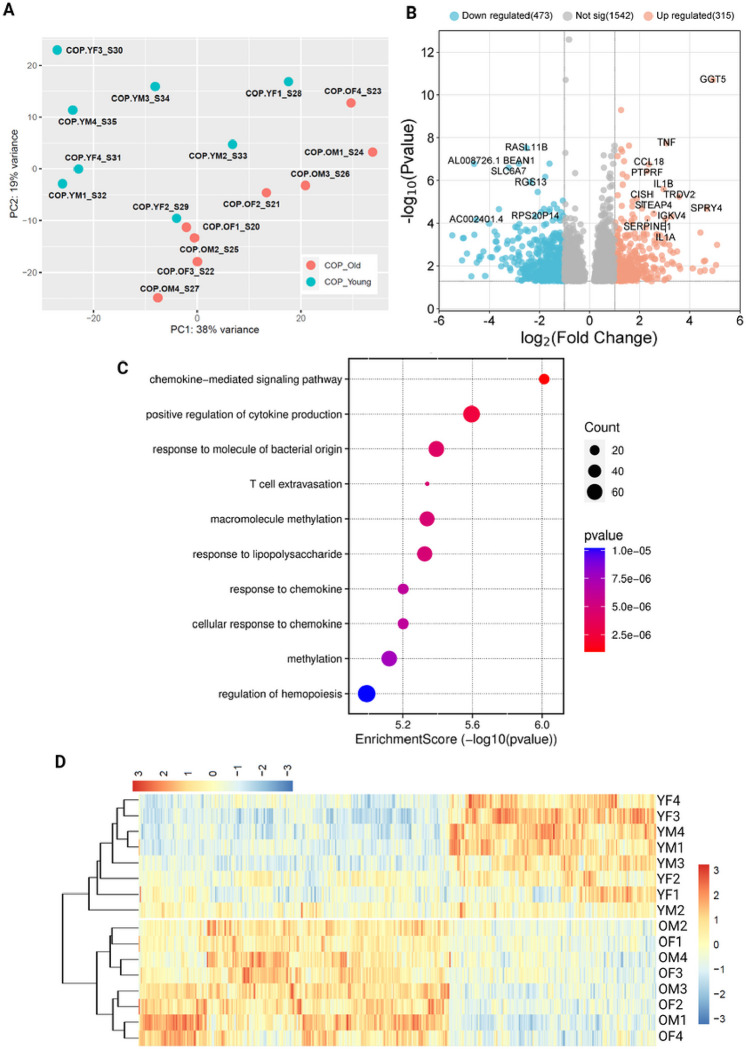
Differential expression of genes across COP cells grouped by age. A) PCA plot showing limited clustering by age, B) Volcano plot showing differential expression of younger samples, compared to older, C) Biological process enrichment analysis, D) Heatmap showing differentially expressed genes. COP: Circulating osteoprogenitor cells; MPC: Mesenchymal stem and progenitor cells; ADASC: Adipose derived adult stem cells; YM: Young Male; YF: Young Female; OM: Older Male; OF: Older Female.

The transcriptome of COP cells showed some level of clustering on PCA, along both PC1 and PC2, however, this was inconsistent, and separation of age groups was poor (Fig. 5A). The transcriptome of COP cells has a large number of differentially expressed genes between older and younger samples, with 473 under-expressed and 315 over-expressed genes in younger individuals vs older people (Fig. 5B and D). Biological process ontology analysis showed enrichment in several pathways associated with immune system activation and reactivity, as well as macromolecule methylation (Fig. 5C). Despite the large number of DEGs, there were no consistently regulated proteins in the older COP cell samples vs the younger.

**Figure 6:**
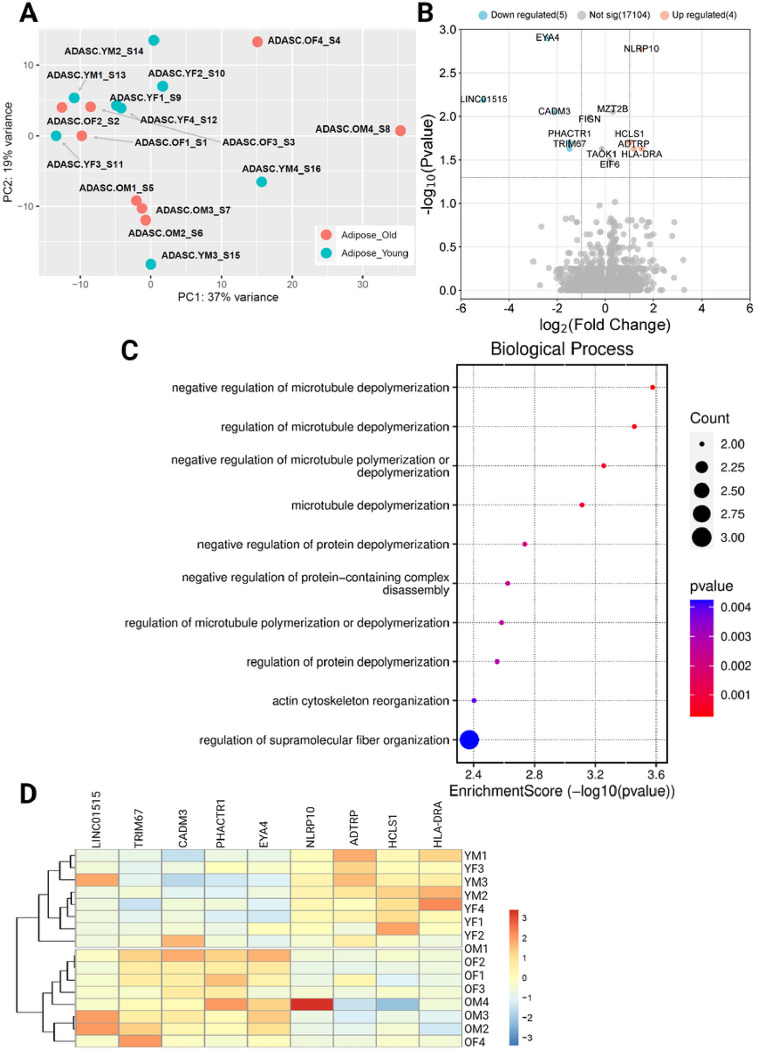
Differential expression of genes across ADSCs grouped by age. A) PCA plot showing no clustering by age, B) Volcano plot showing differential expression of younger samples, compared to older, C) Biological process enrichment analysis, D) Heatmap showing differentially expressed genes. COP: Circulating osteoprogenitor cells; MPC: Mesenchymal stem and progenitor cells; ADASC: Adipose derived adult stem cells; YM: Young Male; YF: Young Female; OM: Older Male; OF: Older Female.

The transcriptome of ADSCs also showed no clear clustering on PCA by age (Fig. 6A), and there were far fewer differentially expressed transcripts, with 5 under-expressed and 4 over-expressed in younger donors vs older donors (Fig. 6A and 6D). Biological process ontology analysis of the DEGs showed largely pathways related to cytoskeletal rearrangement were enriched across the sample. As with COP cells, there were no differentially expressed proteins between the younger and older samples.

## Discussion

For mesenchymal progenitor populations to realize their potential for clinical utilization, a solid understanding of how their physiology changes over the life span is critical. In this work, we compared the transcriptome and proteome of primary MPCs, COP, and ADSCs from older adults to that of younger adults, to identify key mechanisms of stem cell aging. Across all samples, there were a greater number of changes seen in the transcriptome, but broadly, these did not carry through into the proteome of the cells, except for in the MPCs. Only older MPCs had a distinctive proteomic phenotype with several differentially regulated proteins, which may have clinical implications.

The primary goal of this work was to identify whether there were overarching transcriptomic or proteomic changes that typified an older stem and progenitor cell, which may serve as a therapeutic or prognostic target in the management of key musculoskeletal diseases associated with aging. Interestingly, the transcriptome of the pooled stem cells did show a set of consistent changes across the older progenitor cells, irrespective of cell type. The only gene under-expressed in older adults compared to younger ones was the zinc finger and BTB domain containing 16 (ZBTB16), a nuclear transcription factor involved in regulating cell cycle progression, which is a key regulator of stem cell self-renewal and differentiation ^20^. A decrease in this gene may underpin several alterations in differentiation seen in stem cells from older adults. It has been shown that ZBTB16 is highly expressed in undifferentiated stem cells with high capacity for proliferation, and then downregulated as a stem cell becomes terminally differentiated ^20^. In addition, ZBTB16 has been shown to be a key regulator of osteoblastic differentiation in MPCs, again suggesting its suppression could lead to altered regeneration of bone ^21^.

Additionally, there was a significant decrease in melanotransferrin (MELTF) expression in older stem cells across all three groups. MELTF (also known as CD228) is a membrane-bound transferrin, first associated with melanoma development. More recently, it has been found to be expressed in bone marrow MPCs, and interestingly, its inhibition was associated with both increased adipo- and osteogenesis ^22^. There was also an increase in the expression of the Ras-related protein RAB3A, plakophilin 2 (PKP2) and NOTCH-regulated Ankyrin Repeat Protein (NRARP) genes, as well as the MTND1P23 pseudogene. The RAB3A and NRARP genes have never been clearly associated with mesenchymal progenitor function. RAB3A is a key mediator of neuronal exocytosis of neurotransmitters, and has been well studied in neurological tissues, and it is unclear what its role in mesenchymal progenitor may be ^23^. There is however significant cross over seen between neuronal, and mesenchymal progenitors from a number of sources ^24–27^. The NOTCH signaling pathways have a substantial role in stem cell differentiation, associated with muscle homeostasis ^28^, bone formation ^29^, and adipogenesis ^30^.In muscle tissue, while NRARP is upregulated in response to NOTCH signaling, it is a negative regulator – reducing the ultimate formation of tissue ^31^. PKP2 is associated with cardiac muscle cell differentiation in normal contexts, mutations in the gene causing decreased contractility and desmosomal structure in arrhythmogenic cardiomyopathy ^32^, however, it has never been evaluated in mesenchymal progenitors. While many of these transcriptomic changes suggest potential effects on physiologies tied to stem cells, it is notable that none of these were carried through to the proteome. This may be due to the critical role of local environmental factors in the stem cell niche, which drive a significant portion of stem cell behavior ^33^. Throughout fetal, infant and adolescent development, the stem cell niche and the progenitors co-regulate, leading to the development of the distinct populations of adult stem cells ^33^. It is possible that while there may be underlying genetic changes contributing to the decline in stem cell function, the disparity in the environment of bone marrow MPCs, COP cells, and ADSCs leads to different phenotypes of expressed protein.

When analyzed alone, only the bone marrow MPCs showed significant changes in both the transcriptome and proteome. The most notable changes seen in the transcriptome of the MPCs were in genes associated with steroid hormone response and muscle differentiation. The genes related to steroid hormone response over-expressed in older stem cells were Peptidyl Arginine Deiminase, Type II (PADI2) and SET and MYN-domain containing 3 (SMYD3). PADI2 has a role in the maintenance of stem cell proliferation and differentiation, with increased expression leading to greater proliferation, possibly suggesting promotion of stem cell function ^34^. However, overexpression of PADI2 in bone marrow MPCs leads to increased interleukin-6 (IL6) expression, a key inflammatory mediator ^35^. In recent years, the immune role of bone marrow MPCs has gained prominence, and it is now known that they contribute, and in turn are influenced by the chronic inflammation associated with aging ^36^. This overexpression of PADI2 could represent either a mechanism driving proinflammatory change, or a response to chronic inflammation in the bone marrow. SMYD3 is a chromatin-modifying oncogene overexpressed in cancer stem cells, where it promotes proliferation, regulates the cell cycle, and mediates immortalization of cancer cells ^37^. In development, over-expression of SMYD3 regulates mesodermal tissue fate of embryonic stem cells ^38^, however, little is known about its role in adult mesenchymal progenitors. In addition to the over-expression of SMYD3 and PADI2, both the period circadian regulator 1 (PER1) and Alkaline Phosphatase, biomineralization associated (ALPL) genes were under-expressed in older cells. ALPL, the subvariant of alkaline phosphatase associated with the bone, liver, and kidney, is strongly associated with osteogenesis and mineralization in MPCs, and its under-expression is highly suggestive of reduced capacity for osteoblastic differentiation ^39, 40^. In addition, ablation of the ALPL gene in MPCs induces characteristics of bone aging, including impaired osteogenesis, and lipid accumulation, further strengthening its mechanistic role ^39^.The role of PER1 in aging MPCs is less clear. PER1 is one of the most important mechanistic drivers of the circadian rhythm, being cyclically expressed on an approximately 24-hour cycle in the suprachiasmatic nucleus, where it is a master regulator of chronobiology in a range of tissues throughout the body, affecting sleep wake cycle, appetite and energy metabolism, and cell cycle control ^41^. However, PER1 has also been shown to have diverse roles in the physiology of several stem cells, including embryonic, ADSC, MPCs, and dental pulp stem cells ^42^. In mesenchymal progenitor populations, it has been shown to have significant effects on the differentiation of the cells, with osteoblastic, chondrogenic and adipogenic capacity affected by expression of PER1, or its downstream factors such as BMAL1 ^43^, with PER1/BMAL1 knockout animals having increased bone volume ^44^.

While there is a well-known decrease in muscle anabolism in older adults, counterintuitively, the four differentially expressed genes associated with muscle cell differentiation Gremlin1 (GREM1), SMYD3, Delta/Notch-like epidermal growth factor (EGF)-related receptor (DNER), and epiregulin (EREG), were all overexpressed in older MPCs. The direct role of bone marrow MPCs in muscle repair and regeneration is controversial. While bone marrow MPCs can differentiate to form skeletal muscle, this is unlikely to occur directly *in vivo*, with muscle having a dedicated reserve of lineage-restricted satellite cells which serve to regenerate and repair tissue ^45^. However, while in satellite cells, these genes are associated with muscle development, GREM1 expression also identifies stem cells in bone, promoting osteoblastogenesis via RUNX2 expression ^46^, and DNER promotes proliferation through PI3K/AKT signaling in cancer stem cells ^47^, suggesting other potential roles for the genes outside skeletal muscle.

At the proteomic level, the most prominent ontology represented in the differentially expressed proteins was negative regulation of growth, with the staniocalcin1 (STC1), Semaphorin7a (SEMA7A), and NOTCH2 proteins all overexpressed in older MPCs. STC1 is an anti-apoptotic, anti-oxidative protein, secreted by MPCs in response to inflammation, where it inhibits the NLRP3 inflammasome ^48^. STC1 enhances stem cell survival by reducing oxidative stress, and its expression in the older primary cells is likely a response to inflammation rather than the cells themselves being inherently anti-inflammatory compared to younger cells ^48, 49^. Overexpression of SEMA7A in MPCs has also been linked to the inflammatory and oxidative stress response, promoting the secretion of the key anti-inflammatory interleukin 10 from resident macrophages ^50^. NOTCH2 is a well-known factor involved in both immune and musculoskeletal tissue development. Increased NOTCH2 expression has been shown in MPC isolated from geriatric mice, in whom there was greater adipogenic, and poorer osteogenic differentiation ^51^. It has also been demonstrated that MPCs express NOTCH2 to induce the accumulation of regulatory dendritic cells (DCs) in order to curb inflammation in response to lipopolysaccharides ^52^. Taken together, these genes appear to indicate that MPCs from older individuals are under greater oxidative and inflammatory stress, which is then likely to impact terminal differentiation and tissue formation.

The changes to expression in COP cells were less clear than in the bone marrow MPCs, with a large number of differentially expressed genes in the transcriptome but no consistent proteomic phenotype. The clear change to the transcriptome of COP cells in older adults is a shift to pro-inflammatory signaling, with overexpression of key inflammatory mediators such as interleukin 1 (IL1)-beta, IL1-alpha, and tumor necrosis factor, as well as important chemotactic factors CCL18, and GGT5. COP cells have been shown to have a more prominent immune role than bone marrow MPCs, and ADSCs ^53^, and thus it stands to reason that they may be more strongly affected by inflammaging and chronic oxidative stress with advancing age. In addition, given their native environment in the circulation, they are likely exposed to a wide range of factors reflective of the general condition of the individual. Given the well-known increase in chronic inflammation in older age, it is likely that COP cells are regulated into a pro-inflammatory state, and then in turn drive further chronic inflammation. Whether this leads to direct impacts on musculoskeletal tissues as COP cells home to locations such as bone is unknown. Like COP cells, the bone-resorbing osteoclast is a monocyte lineage cell sensitive to inflammation, which drives osteoclastogenesis and catabolism of mineralized bone. It has been suggested that COP cells may mediate a link between the bone and immune systems ^9, 10^, regulating both osteoclast and osteoblast within the bone microenvironment, and the pro-inflammatory shift seen in the older cells in this study may drive the acceleration of bone loss in aging. The diversity in the microenvironment of COP cells in different populations may also explain the lack of a consistent proteomic phenotype. Significant external stimulus input leads to a large amount of post-translational modification and regulation, likely leading to considerable variance in the ultimately expressed proteome of the cells.

A similar pattern of expression was seen in the ADSCs, with a small number of differentially expressed genes, but no consistent proteomic phenotype in the older cells. Most changes in the transcriptome were centered on regulation of the cytoskeleton, immune response initiation and cell movement and adhesion. Older ADSCs had increased expression of the LINC101515 noncoding RNA, as well as CADM3, PHACTR1, TRIM67, and EYA4. These genes have not been evaluated in mesenchymal progenitors, and so the significance of these changes is unclear. However, increased expression of PHACTR1 has been shown to increase mineralization within endothelial progenitors ^54^, and is known to be involved in cell mobility, apoptosis, and matrix remodeling ^55^, though whether this is the case in ADSCs requires further research. As with COP cells, there was also no evident phenotype in the proteome of the ADSCs. This may again be due to the variety of changes accruing in the vascular microenvironment in the adipose tissue of older adults, based on health status. Adipose tissue strongly regulates and is in turn regulated by a range of chronic diseases, and the profile of the local stem cells is likely to reflect this.

### Strengths and Limitations

The major strength of this study is its scale – there is no other comparable work using primary harvested stem cells, across as many individuals, providing significant power to detect changes across the data set. In addition, robust data management, cell treatment and analysis, with all samples grown and analyzed simultaneously provide confidence in the findings. The major challenge with in vivo generalizability in these results is the expansion of the cells in culture. In an ideal setting, cells would be harvested and analyzed directly, however due to the scarcity of the cells, expansion is required to get significant volumes of protein and RNA. Expansion was minimized to ensure the smallest amount of variability between the data gained here and what is likely the case in vivo. In addition, the lack of broader health information from the donors makes some of the changes, or lack thereof, difficult to contextualize. All donors were cleared of major disease prior to cell isolation, however addition of general health indicators, or biomarkers of inflammation, metabolic disorders, or other key physiological indicators associated with disease would improve generalizability.

## Conclusion

In this study, we show a range of mechanistic changes occurring with the aging of mesenchymal stem cells. This understanding will have important implications for ongoing work in stem cell therapeutics in musculoskeletal disease of older adults, allowing for improved screening, stimulation, and individualization of treatments. Of note is the pro-inflammatory shift of stem cells taken from older adults and a pattern of altered differentiation status in all three stem cell types. This has significant implications for treatment of specific diseases, for example, COP cells harvested from older adults may not be suitable in the context of inflammatory diseases such as osteoarthritis, and autologous MPCs may not be ideal for muscular applications. These results should allow for greater individualized treatments for both disease and patient, improving outcomes. Future research should build on this work by investigating the influence of chronic disease on the physiology of stem cells in older adults, as well as exploring the roles of the identified differentially expressed genes and proteins from this study, many of which have not been adequately explored in mesenchymal progenitor populations.

## Methods

### Biosafety and ethics

This study was undertaken in laboratories at the Medical University of South Carolina (MUSC), SC, USA, and the University of Melbourne, Victoria, Australia. All experiments were undertaken with aseptic procedures, under appropriate biosafety conditions. All human samples were collected following Human Research Ethics Approval at the site of collection. The COP cell samples were acquired from the Australian Red Cross Blood Service (ARCBS), following a waiver of ethics requirements from the Western Health Human Research Ethics Committee.

#### Primary cell cultures:

The experiments in this study were undertaken on primary human musculoskeletal progenitor cells taken from donors from specific age and sex groups. For each primary cell type (COP, ADSC, and MPCs), there were 16 total samples – four taken from younger (18–40) males, four from younger females, four from older (50–80) males and four from older females, for a total of 48 samples which underwent analysis. Samples from each of the 48 donors underwent both transcriptomics and proteomics, to allow more accurate alignment of the datasets.

##### COP cells:

COP cells were acquired as documented in a previous validation study ^56^. Briefly, buffy coats were acquired from therapeutic blood donations from the ARCBS, and white cells purified through Ficoll density gradient separation (GE Healthcare Companies, GE17–1440-02). Once purified buffy coats were collected, fluorescence activated cell sorting was used to isolate the COP cells. The isolated leukocytes were incubated with FcR blocking reagent (Miltenyi Biotec, Cat. No: 130–059-901) for 10 minutes, before being labelled with the conjugated fluorescent antibodies ((CD45- Fluorescein isothiocyanate (BD Biosciences, Cat. No: 555482), ALP-brilliant violet 421 Allophycocyanin (BD Biosciences, Cat. No: 752998), and CD34-Allophycocyanin (BD Biosciences, Cat. No: 340441)) at a concentration of 1:100 v/v in the dark at 4 degrees Celsius (°C) for 30 minutes. Cells were then co-stained with a viability dye (7-AAD [BD-Biosciences, Cat. No: 559925]) and processed on a four laser (405 nm, 488 nm 561 nm, and 633 nm) FACSAria III flow cytometer, and collected on ice in sterile tubes. COP cells were defined as the CD45+/CD34+/ALP + population during flow cytometry. The COP cells were then plated at a density of 1.25×10^5^ on fibronectin coated tissue culture flasks in low-glucose Dulbecco’s modified eagle medium (DMEM), supplemented with 15% FBS, 1% penicillin/streptomycin and 2.5 mM l-glutamine. The cells underwent two passages until adequate cells had been collected for the transcriptomic and proteomic analyses.

### MPCs

5–8 ml of red bone marrow was collected via aspiration from the vertebral bodies of orthopedic surgery patients at MUSC under the approval of the Institutional Review Board. The bone marrow was collected into EDTA coated tubes, then passed through a 100 μm filter to remove bone debris, and cell aggregates. The bone marrow then underwent density gradient separation before incubation with a magnet conjugated anti CD271 antibody for 30 minutes at room temperature. The cells were then passed through a magnetic column with the CD271 + MPCs isolated as characterized previously ^57^. The MPCs were then cultured in DMEM with the same supplements described above. For two passages, until adequate cell numbers had been acquired.

### ADSCs

Primary ADSCs from liposuction aspirated were acquired commercially for research use (LACell, New Orleans, LA) as previously described ^58^. Only samples from Non-Obese, healthy donors were used in the analyses. The ADSCs were thawed into DMEM as described above and passaged twice to gain adequate cell numbers for the analysis.

#### Transcriptomic analyses:

##### RNA Isolation

Following two passages in cell culture, COP cells, MPCs and ADSCs were washed twice with PBS, trypsinized, before undergoing RNA extraction with the QIAGEN miRNEasy Minikit (QIAGEN, USA) according to manufacturer instructions. RNA purity and integrity was evaluated by Qubit fluorometer (Invitrogen, USA), and Agilent TapeStation electrophoresis, with all RNA used in the analysis having a RIN > 9. Secondary assessment of RNA integrity was performed with AATI fragment analyzer to ensure no degradation in processing and transport.

##### RNA sequencing

Sequencing was performed at the Micromon genomics institute at Monash University, Melbourne, Australia. Libraries were prepared with the MGIEasy RNA chemistry system, and sequencing performed on an MGITech MGISEQ2000RS system. Sequencing was performed over three sequencing lanes, with greater than 400 million raw reads per lane. Reads were mapped to the human genome index file from the University of California, Santa Cruz, (March 2021) with the ‘Rsubread’ package on R (v4.0.5). Phred scores were calculated, with scores > 30 deemed adequate for analysis.

###### Proteomics:

Second passage cells were collected by trypsinization and washed three times with PBS to remove contaminant protein. Cells were then lysed by agitation in a 9 M urea, 50 mM Tris-HCl, with 100 units/ml of nuclease at pH8 for 30 minutes then centrifuged at 20,000×g for 15minutes. Protein was then reduced in dithiothreitol (1 mM), and alkylated in iodoacetamide (5 mM), and digested with Lys-C at a 1:50 ratio of protease to protein for 3 hours, then overnight in trypsin at 37°C at the same ratio. The digestion was then acidified with to 1% with formic acid and desalted with C18 stage tips conditioned with 5% formic acid, and 80% acetonitrile, before being dried in a SpeedVac.

###### Mass Spectrometry:

The purified peptides were analyzed via label free proteomics on an EASY nLC 1200 System (ThermoScientific) in-line with the Orbitrap Fusion Lumos Tribrid Mass Spectrometer (ThermoScientific) (control software v. 4.2.28.14). Two μg of peptides were loaded on C18 reversed-phase column (Acclaim PepMap RSLC, 75 μm × 50 cm (C18, 2 μm, 100 Å) ThermoFisher cat. # 164536) using a 5–40% B gradient in 180 minutes (Solvent A: 5% acetonitrile/ 0.1% formic acid; Solvent B: 80% acetonitrile/ 0.1% formic acid) at a flow rate of 300 nL/minute.

Spectra were acquired with a high resolution (60,000) FTMS survey scan in data-dependent mode, with a mass range of m/z 375–1500, followed by 3s cycle time tandem mass spectra (MS/MS) of the most intense precursors. HCD fragmentation was performed with a precursor isolation window of 1.6 m/z, a maximum injection time of 50ms, and HCD collision energy of 35%. Precursors within 10 ppm mass tolerance were dynamically excluded from resequencing for 15 sec. Precursor ions with charge states that were undetermined, 1, or > 5 were excluded.

##### Mass Spec processing

The spectra acquired were searched through the MaxQuant platform (v.1.6.3.3) and normalized with the label free quantification (LFQ) algorithm. Data was matched to the SwissProt database (March 2021), and a database of contaminants. FDR was calculated through reverse database strategy, set at 1% at protein and peptide level. Peptides were required to be fully tryptic, and of at least 7 residues with lysine-proline cleavage. A maximum of two missed cleavages were permitted. The MaxQuant results then analyzed in Perseus^59^. Proteins identified by only a single modified peptide, contaminants, and reverse matched peptides, were removed and LFQ protein intensities log_2_ transformed. Protein intensities were visualized, and normalized, and ultimately had similar distribution.

### Statistical analysis

Lowly expressed genes were filtered using edgeR, and library sizes and distribution visualized and normalized. Normalization was also performed to limit composition bias. Finally, differential expression analyses for pre-specified contrasts (younger donors, vs older donors) with limma-voom using contrast analysis and moderated Bayesian statistics. Comparisons with a false discovery rate (FDR) < 0.05 were considered significant. Additional pathway analyses and gene ontology analyses were performed to identify functional changes within the datasets using SRPlot ^60^. The raw data for this study is publically available at the Sequence Read Archive with the accession number PRJNA987312 (sequencing data), and the mass spectrometry proteomics data have been deposited to the ProteomeXchange Consortium via the PRIDE partner repository with the dataset identifier PXD035803.
